# CT-optimized skin stroking delivered by hand or robot is comparable

**DOI:** 10.3389/fnbeh.2013.00208

**Published:** 2013-12-18

**Authors:** Chantal Triscoli, Håkan Olausson, Uta Sailer, Hanna Ignell, Ilona Croy

**Affiliations:** ^1^Department of Clinical Neurophysiology, Institute of Neuroscience and Physiology, University of GothenburgGothenburg, Sweden; ^2^Department of Psychology, University of GothenburgGothenburg, Sweden

**Keywords:** C tactile, pleasant touch, robot touch, physiology, nerve fibers, unmyelinated

## Abstract

**Introduction**: The aim of our study was to investigate whether a pleasant tactile stimulation which is manually produced is qualitatively comparable to an analogous tactile stimulation produced instead by a mechanical source.

**Methods**: Thirty-one subjects [16 men, 15 women, 24.5 ± 2.6 years, mean, and standard deviation (SD)] were tested under four different conditions in a repeated measurements design. A pleasant caress-like brush stroke on the hairy skin of the forearm was either produced by a robot or by hand with three different velocities (0.3, 3, and 30 cm/s). In two conditions the subjects were informed about the stroke's source, whereas in two different conditions they were not. Subsequent to the stimulation, the subjects were asked to rate both pleasantness and intensity of each tactile sensation.

**Results**: Consistently, pleasantness ratings were very similar in both conditions. This was found across stimulus velocities and regardless of whether the subjects were informed about the source of the on-going stroke or not. In contrast, intensity ratings were significantly higher in the handheld condition for the two slower velocities, but not for the fastest one.

**Conclusion**: As robot and human touch are highly comparable in terms of perceived pleasantness, handheld stimulation may be used in studies of touch hedonia where robot stimulation is not applicable (for instance in children or certain body parts).

## Introduction

The sense of touch is fundamentally important in everyday life. For human hairy skin pleasant and affective touch can be distinguished from discriminative touch by involving partly different peripheral pathways: pleasant touch perception relies on signaling in a group of unmyelinated, slowly conducting (1 m/s) low-threshold mechanoreceptors, called C-fiber tactile (CT) afferents. (Olausson et al., 2008, 2010; Loken et al., 2011; McGlone et al., 2012). On the other hand, discriminative touch is dependent on low-threshold myelinated mechanoreceptors. This class of afferents plays a role in capturing details of objects such as their shapes and textures, in order to perceive complex object features and aid in object manipulation (Olausson et al., 2010).

The optimal stimulus for activation of CT afferents, as indicated by psychophysical and microneurography measurements, was found to be a slow stroke within a velocity range of 1–10 cm/s, which approximately corresponds to the velocity of caress-like touch between individuals (Loken et al., 2009; McGlone et al., 2012). This observation is one of the key observations for the “social/affective touch” hypothesis proposing that CT afferents play a role in eliciting the pleasant subjective experience that accompanies behavioral responses to gentle touch between individuals (Olausson et al., 2010; McGlone et al., 2012).

CT stimulation is usually performed using a brush which is moved with a certain force and velocity over the hairy skin (mostly forearm). This stimulation can be done handheld or with the help of a robot that allows very precise control of stimulus application. In the last years a number of studies explored CT afferent touch by giving handheld (Olausson et al., 2008; Loken et al., 2011; Morrison et al., 2011; Ackerley et al., 2012) and robot stimulation (Blakemore et al., 1999; Olausson et al., 2002; Loken et al., 2009, 2011; Morrison et al., 2011; McGlone et al., 2012). However, it is unclear if handheld and robot stimulations are comparable in terms of pleasant touch perception. We therefore, examined the effect of handheld and robot stroke stimulation on perceived pleasantness. To avoid an eventually different sensory quality of the stimuli affecting the pleasantness ratings, we also asked about the perceived intensity.

Additionally, the influence of knowledge of the experimental setting was examined. It has been shown before that touch ratings can be influenced by expectations (McCabe et al., 2008; Gazzola et al., 2012). When touch stimuli were labeled as “rich cream,” for instance, they were perceived more positively than when they were labeled as “basic cream” (McCabe et al., 2008). It can be hypothesized that humans rate touch in a different way when given by a human, for instance because of social desirability. This phenomenon refers to a bias toward social norms, or toward a behavior which subjects believe the researcher would desire (King and Bruner, 2000; Sanzone et al., 2013). Alternatively, according to the “like me”- theory, actions of entities supposed to be similar to the self are preferred to the actions of entities dissimilar to the self (Meltzoff, 2007). We therefore, hypothesize that the mere knowledge that touch is performed by a human may induce the expectation that it should feel better and accordingly lead to higher pleasantness rating.

To explore those questions, four different conditions were compared in which the participants were stroked by a brush that was either mounted on a robot or handheld (source of stroking) with or without being aware of the source.

## Methods

### Participants

In total, 31 subjects, aged between 20 and 30 years (mean age = 24.5; standard deviation (*SD*) = 2.61) were recruited, 16 of them were men and 15 of them were women. The majority of the participants were students. The study was approved by the ethics committee of the University of Gothenburg and the participants received financial compensation for participation in the study.

After each experiment, all 31 subjects were tested using two different questionnaires: The Becks depression questionnaire, BDI-II (Beck and Steer, 1987) is a validated questionnaire assessing the severity of depression with 21 items and results in a sum scale ranging from 0 to 63. The Tactype (Deethardt and Hines, 1984) assesses the subjective features of social interactions with 15 items and results in a sum scale ranging from 15 to 75. The questionnaires were administered in Swedish for 11 Swedish students and in English for 20 foreign students.

All participants completed both questionnaires [BDI: mean score = 5.8, *SD* = 6.3; *N* = 29 (93.6%) in the range of none to minimal depression, *N* = 2 (6.3%) in the range of moderate depression; Tactype: mean score = 60.2, *SD* = 6.5]. None of the participants was excluded from the study.

### Materials

#### Experimental setting and procedure

After explanation of the experiment (not involving details about the four different conditions) and signing the informed consent form, the participants were asked to sit in a comfortable chair in front of a computer screen, and to put their left arm in prone position on a pillow positioned on the left side of the chair. On the subject's left side, the stimuli were applied to the subject's left dorsal forearm by an experimenter sitting next to the robot. On the subject's right side, a mouse was placed on a table, used for rating the stimuli. The rating scales were presented on the computer screen.

Four different conditions of stroking were given to each participant (Table 1). In the first two conditions, the participants were not informed about the source of stimulation (“not-informed” conditions). They were asked to put on headphones with pink noise and occluding glasses that blocked their peripheral vision to shield them from distracting stimuli and to avoid them seeing the source of stimulation. After those conditions the participants were informed that stimuli had been applied once by robot and once by hand and that in the next two conditions they would be told about the source of stimulation. During the next two conditions (“informed” conditions), the participants were still shielded from auditory and visual distraction, but informed about the source of stimulation before each condition.

**Table 1:** **Experimental design and randomization**.

		**Awareness (not randomized)**				
		**Not informed**			**Informed**	
		**Source (randomized)**				
		**Handheld**	**Robot**	**Handheld**		**Robot**
Velocity (randomized)	0.3 cm/sec	3 repetitions	3 repetitions	3 repetitions		3 repetitions
	3 cm/sec	3 repetitions	3 repetitions	3 repetitions		3 repetitions
	30 cm/sec	3 repetitions	3 repetitions	3 repetitions		3 repetitions

The order of conditions for “hand/robot” stroke was randomly assigned among the participants. Depending on the participants' order of appearance, brushing started either by hand or by robot (both for the first and the informed and the not-informed condition). The stroke was performed through two identical brushes (a 50 mm wide flat, soft watercolor brush made of fine, smooth, goat's hair) in order to avoid any rating variations due to a different texture of the two brushes. In the “robot” condition, the brush strokes were delivered by a custom-built robotic device (rotary tactile stimulator, RTS; Dancer Design, St Helens, UK) driven by LabVIEW (National Instruments, TX) software at a calibrated normal force of 0.4°N. In the “handheld”-condition, the brush was held by the experimenter (Chantal Triscoli). The experimenter was trained in delivering the stimuli in all three velocities and with a constant force by visually controlling the bending of the bristle brushes. Moreover, the robot performed the brushing with the same velocity next to the experimenter (brushing in the air) during the “hand”-condition and thus, guided the hand-held brush strokes. In both the robot and the handheld condition, the brush stroked the forearm for 10 cm in proximo-distal direction. Then it was lifted up and moved back detached from the arm. After each stroke the participant rated the stimulus on two visual analog scales: one for intensity (0 = not intense at all, 10 = extremely intense) and one for pleasantness (−10 = extremely unpleasant, 10 = extremely pleasant). “Intensity” was explained to the participants as the grade to how much they perceive the stroke. The inter-stimulus interval was set to 15 s.

For all four conditions (informed/not informed ^*^ robot/ handheld), three different brush-stroke velocities were used: “fast” (30 cm/s), “slow” (3 cm/s), and “very slow” (0.3 cm/s), with 3 repetitions each. Consequently, 9 strokes were presented per experimental condition. The three velocities differ in experienced pleasantness and the degree of CT fiber activation. CT fibers of hairy skin (forearm) respond most vigorously to slow and light stroking with a velocity between 1 and 10 cm/s, corresponding to our “slow” velocity (Loken et al., 2009). The velocities and the source of stimulation were randomized within and across the participants (compare Tables 1, 2).

**Table 2:** **Randomization matrix**.

**Randomization of sequences**					**Randomization of velocities within the sequences**									
**Participant-Nr**.	**Not informed**		**Informed**		**Randomization-Nr**.	**Velocity in cm/s**								
	**Seq 1**	**Seq 2**	**Seq 3**	**Seq 4**	1	0.3	3	30	3	3	30	0.3	0.3	30
1, 8, 15, 22, 29	H1	R1	H2	R2	2	3	30	3	0.3	0.3	30	30	3	0.3
2, 9, 16, 23, 30	R2	H2	R3	H3	3	30	30	3	0.3	3	0.3	30	0.3	3
3, 10, 17, 24, 31	H3	R3	H4	R4	4	3	3	30	30	0.3	3	0.3	30	0.3
4, 11, 18, 25	R4	H4	R5	H5	5	3	30	0.3	0.3	30	0.3	3	30	3
5, 12, 19, 26	H6	R6	R7	H7	6	0.3	30	30	3	0.3	3	30	3	0.3
6, 13, 20, 27	R7	H7	H8	R8	7	0.3	3	30	30	3	0.3	3	0.3	30
7, 14, 21, 28	H8	R8	R1	H1	8	30	0.3	3	0.3	3	0.3	3	30	30

H1 … handheld presentation of stimuli randomized according to the randomization-nr. 1.

R1 … robot presentation of stimuli randomized according to the randomization-nr. 1.

### Statistical analysis

First, the three repetitions of each intensity and pleasantness VAS-rating per velocity/condition were averaged. These values were then used for further statistical analysis. All statistical analyses were made using SPSS version 21 (IBM, Chicago, USA). The main effects of velocity (fast, slow, very slow), source (robot, handheld) and awareness (aware, not aware) were analyzed using two separate ANOVAs (3^*^2^*^2) for repeated measurements, one with pleasantness and one with intensity as dependent variable. The effects of age and gender were analyses by adding age as a covariate or gender as a between subject factor into the analysis.

Afterwards, the effects of source and awareness were analyzed for each of the three velocities separately using ANOVAs for repeated measurements [source (2)^*^ awareness (2)] and are reported with a Bonferroni-correction using the factor three, reflecting the number of tests for three velocities. Greenhouse-Geisser correction was used to adjust for violations of sphericity. Level of significance was set to 0.05.

## Results

**Figure 1:**
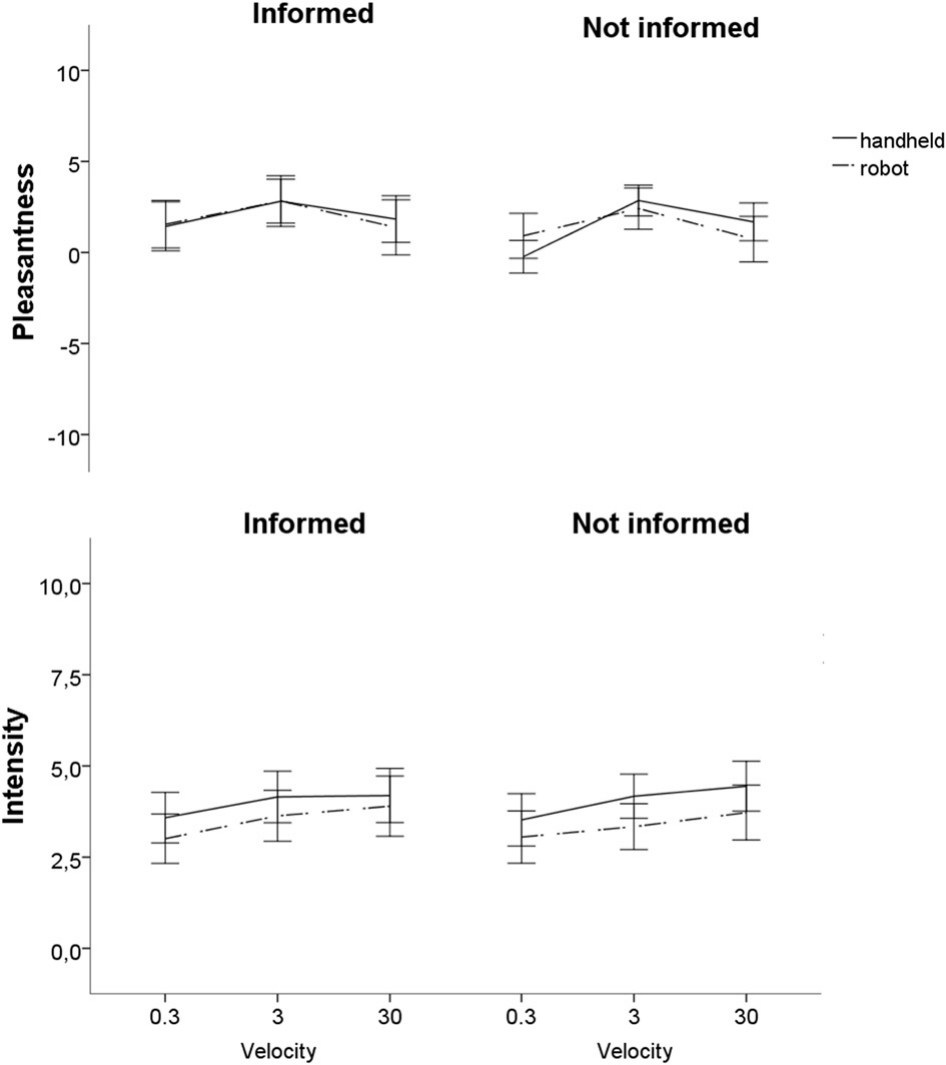
**Pleasantness and Intensity ratings in handheld and robot touch**. There was no significant main effect of source on pleasantness rating, but on intensity (*p* < 0.001). Note: Error bars represent the 95% confidence intervals.

Velocity of the brushing had a significant main effect on the ***pleasantness rating*** [*F*_(2, 60)_ = 8.4, *p* = 0.03, compare Table 3 and Figure 1]. Pairwise comparisons revealed that stimuli presented with 3 cm/s led to significantly higher pleasantness ratings than stimuli presented with 0.3 or 30 cm/s (each *p* < 0.001). There was no significant difference in pleasantness between stimuli presented with 30 and 0.3 cm/s. There was no significant main effect of awareness or of the source of stimulation on the perception of pleasantness. However, there was an interaction between the source delivering the stimulus and the velocity [*F*_(2, 60)_ = 5.6, *p* = 0.01]. Participants preferred the handheld over the machine stimulus at the velocity of 30 cm/s and vice versa at the velocity of 0.3 cm/s. However, those results were not significant after Bonferroni correction. Furthermore, there was a significant interaction between awareness and velocity [*F*_(2, 60)_ = 5.8, *p* = 0.006]. *Post-hoc* testing revealed that the informed condition tended to lead to higher ratings of pleasantness at the velocity of 0.3 m/s [*F*_(1, 30)_ = 5.4, *p* = 0.08], but not for the other velocities. There were no other significant interaction effects and there were no significant effects of age and gender on the pleasantness ratings.

**Table 3:** **Mean values of the ratings generated at the different velocities and conditions**.

	**Awareness**	**Velocity in cm/s**	**Source**			
			**Handheld**		**Robot**	
			**Mean**	***SD***	**Mean**	***SD***
**Pleasantness**	Not informed	0.3	0.2	2.5	1.4	3.5
		3	3.5	2.4	3.0	3.2
		30	2.2	2.9	1.2	3.5
	Informed	0.3	1.4	3.7	1.6	3.6
		3	2.8	3.3	2.8	3.8
		30	1.8	3.5	1.4	4.1
**Intensity**	Not informed	0.3	**3.5**	2.0	**3.1**	2.0
		3	**4.2**	1.7	**3.3**	1.7
		30	4.4	1.9	3.7	2.0
	Informed	0.3	**3.6**	1.9	**3.0**	1.8
		3	**4.2**	1.9	**3.6**	1.9
		30	4.2	2.0	3.9	2.2

There was a significant main effect of velocity on pleasantness (p = 0.03) and intensity (p = 0.008). There was a significant main effect of source on the intensity ratings. Post-hoc testing showed that handheld touch was perceived as more intense than robot in strokings applied with 0.3 and 3 cm/s (p < 0.001).

For ***intensity ratings***, there was a significant main effect of velocity [*F*_(2, 60)_ = 7.3, *p* = 0.008] and source (*p* < 0.001, *F* = 15.9), but not of awareness (compare Table 2, Figure 1). The main effect of velocity showed that the higher the velocity, the higher the intensity ratings. Bonferroni correction for pairwise comparisons showed significant differences in intensity ratings between 0.3 and 3.0 cm/s (*p* = 0.001) and between 0.3 and 30 cm/s (*p* = 0.03), but not between 3 and 30 cm/s. *Post-hoc* testing for the main effect of source showed that the handheld stimulus generated significantly higher intensity ratings at 0.3 cm/s [*F*_(1, 30)_ = 12.2, *p* = 0.006] and at 3 cm/s [*F*_(1, 30)_ = 24.3, *p* < 0.001] but not at 30 cm/s [*F*_(1, 30)_ = 4.9, *p* = 0.1] (compare Table 3, Figure 1). No significant interactions were found between the factors awareness, velocity and source and there were no significant main effects of age and sex. A significant interaction between sex and velocity was found [*F*_(2, 29)_ = 6.6, *p* = 0.01]. However, *post-hoc* tests revealed no significant sex difference for any of the three velocities, separately.

No significant correlations were found between the ***questionnaires*** and any of the pleasantness or intensity ratings.

## Discussion

The aim of the study was to establish if human and robotic tactile stimulation lead to a comparable degree of experienced pleasantness. Our results supports this; human and robot touch led to similar pleasantness ratings for CT optimized and non-optimized skin stroking, irrespective of whether participants were aware of the source. However, handheld stroking was perceived as being more intense for the very slow (0.3 cm/s) and slow (3 cm/s) velocities.

Our findings replicate earlier observations of an inverted u-shaped relationship between stroking velocity and hedonic ratings with the highest pleasant ratings being obtained with a velocity of 3 cm/s. Skin stroking with this velocity was reported to be optimal for the activation of CT fibers (Loken et al., 2011; McGlone et al., 2012).

Previous studies have shown that optimal CT stimulation can be obtained through both handheld and mechanical stimulation (Blakemore et al., 1999; Olausson et al., 2002, 2008, 2010; McCabe et al., 2008; Loken et al., 2009, 2011; Morrison et al., 2011; Ackerley et al., 2012; McGlone et al., 2012). Our study suggests that there are no robust differences in the *degree of perceived* pleasantness, which thus, remains unaffected of the delivery by a human or a machine.

Contrary to our hypothesis, there were even no differences in the pleasantness perception of touch if the participants were aware of the source of stimulation. These results are in contrast to findings demonstrating that expectation can modulate the representation and the affective value of the sense of touch when the touch is applied by stroking with the fingers (McCabe et al., 2008). We believe that the contradictory results can be explained by the experimental setting: in our study the physical characteristics of handheld and mechanical strokes were very similar (similar pencil, same velocity, visual, and auditory shielding), which may prevent strong top down influences. Support for this notion comes from a study where static touch was used to modulate the processing of pictures (Schirmer et al., 2011). Although being touched significantly changed event related potentials evoked by pictures, there were no significant differences between touch provided by a friend, by a machine but attributed to the friend, or provided by and attributed to a machine.

Emotional and social components of touch are inseparable from physical sensations (Hertenstein et al., 2006), and the intensity ratings give some guidance about those. In contrast to the inverted u-shape obtained for pleasantness ratings, we found a linear relationship between velocity and intensity ratings. This could be explained by the degree of A beta fiber activation. For those myelinated fast conducting fibers, a linear relationship between firing frequency and stimulus velocity has been shown (Loken et al., 2009). We therefore, speculate that touch intensity ratings are related to A beta fiber activation while pleasantness ratings are related to C-tactile activation.

We found an influence of the source of stimulation on intensity ratings. Handheld touch was rated as being more intense for the slower velocities of 0.3 and 3 cm/s. Special care was taken to deliver the handheld touch exactly: it was led by the robot in terms of speed and the experimenter controlled the pencil hair bending, which is an indicator for the force. However, when the same force has to be preserved for several seconds, the robot stimulation is superior in terms of precision. This difference seems to be perceivable by the participants.

## Conclusion

In a controlled experimental setting, the source of a CT optimized skin stroking produced either from human or mechanical entities led to a very similar sensation experienced by the individuals. Results from studies using one or the other method seem comparable if pleasantness is the main dependent variable. For intensity ratings, however, the less controllable handheld stimulation may be a disadvantage.

Our results indicate that handheld stimulation is a sufficient method to deliver CT optimized stimuli. This is of special interest in cases where robot stimulation cannot be performed, such as studies with young children, certain patient groups, or on smaller body areas.

Furthermore, our results together with results from another group (Schirmer et al., 2011) show that certain aspects of tactile experiences are relatively independent of attribution. Irrespective of the source and the attribution of the source, the CT optimized touch was perceived as significantly more pleasant than the non-optimized. This implies that affective aspects of stroking are rather stable and that the rewarding value of affective touch is relatively robust against top down mechanisms.

### Conflict of interest statement

The authors declare that the research was conducted in the absence of any commercial or financial relationships that could be construed as a potential conflict of interest.
